# Effects of Dietary Microbial Muramidase on the Growth, Liver Histoarchitecture, Antioxidant Status, and Immunoexpression of Pro-Inflammatory Cytokines in Broiler Chickens

**DOI:** 10.3390/ani13243862

**Published:** 2023-12-15

**Authors:** Anaam E. Omar, Ghada I. Abd El-Rahman, Ahmed Gouda, Abdel-Wahab A. Abdel-Warith, Elsayed M. Younis, Samar A. Abdo, Azhar Eltanahy, Ahmed Said Kamal, Simon J. Davies, Shimaa A. Amer

**Affiliations:** 1 Department of Nutrition and Clinical Nutrition, Faculty of Veterinary Medicine, Zagazig University, Zagazig 44511, Egypt; 2 Department of Clinical Pathology, Faculty of Veterinary Medicine, Zagazig University, Zagazig 44511, Egypt; 3 Animal Production Department, Agricultural & Biological Research Division, National Research Center, Dokki, Cairo 11865, Egypt; 4 Department of Zoology, College of Science, King Saud University, P.O. Box 2455, Riyadh 11451, Saudi Arabia; 5 Biochemistry Department, Faculty of Veterinary Medicine, Zagazig University, Zagazig 44511, Egypt; 6 Department of Animal Wealth Development, Faculty of Veterinary Medicine, Mansoura University, Mansoura 35516, Egypt; 7 Department of Birds and Rabbit Medicine, Faculty of Veterinary Medicine, University of Sadat City, Sadat City 32897, Egypt; 8 Aquaculture Nutrition Research Unit ANRU, Carna Research Station, Ryan Institute, College of Science and Engineering, University of Galway, H91V8Y1 Galway, Ireland

**Keywords:** broiler chickens, microbial muramidase, growth performance, antioxidant enzymes, interleukin 1 beta, liver enzymes

## Abstract

**Simple Summary:**

Feeding approaches to increase production efficiency using new feed additives are essential for sustainable poultry production. This study aimed to evaluate the dietary addition of microbial muramidase (MMUR) on broiler chickens’ nutritional and health aspects. The study suggested that MMUR could enhance the chicken growth rate and improve their antioxidant, inflammatory, and anti-inflammatory responses.

**Abstract:**

The impact of microbial muramidase (MMUR) addition to broiler chicken rations was evaluated through growth parameters, liver histoarchitecture, antioxidant status, biochemical analysis, and expression of pro-inflammatory cytokines for 35 days. Four hundred three-day-old chicks (97.68 ± 0.59 g) were distributed to four distinct groups with ten duplicates each (100 chicks/group) consisting of: group 1 (G1): a basal diet without MMUR (control group); G2: a basal diet + 200 mg MMUR kg^−1^ G3: a basal diet + 400 mg MMUR kg^−1^; and G4: a basal diet + 600 mg MMUR kg^−1^. The results showed that the final body weight and total weight gain were increased (*p* = 0.015) in birds fed with diets supplemented with MMUR at 600 mg kg^−1^. The feed conversion ratio (FCR) was improved in all treatment groups compared with the control group. Birds fed with a diet supplemented with 600 mg MMUR kg^−1^ showed the highest body weight gain and improved FCR. The values of thyroxin hormones and growth hormones were increased in all MMUR-supplemented groups. Dietary MMUR increased the activities of antioxidant enzymes (total antioxidant activity, catalase, and superoxide dismutase) and decreased the activity of malondialdehyde (*p* < 0.05). In addition, it increased the values of interleukin 1 beta and interferon-gamma compared with the control group. Furthermore, dietary MMUR increased the expression of transforming growth factor-beta immunostaining in the liver and spleen tissues. Our results show that supplementing broilers’ diets with 600 mg MMUR kg^−1^ could enhance the chicken growth rate and improve their antioxidant, inflammatory, and anti-inflammatory responses.

## 1. Introduction

The addition of exogenous enzymes to broiler chicken rations is a public practice in nutrition, as it helps in digestion and destroys the antinutritional compounds in the feeds, thus improving their nutritional value and the birds’ performance [1], while also minimizing the feed costs and environmental pollution [2,3]. Almost all market feed enzymes target specific substances in the feed ingredients (e.g., carbohydrates, phytases, and proteases) [2]. However, microbial muramidase (MMUR) is considered one of the enzyme categories that target constituents existing in the lumen of the intestine [4,5].

Muramidase, recognized as N-acetylmuramidase or Lysozyme, is an antimicrobial enzyme that forms a portion of an animal’s innate immune response. It occurs naturally in many body fluids and secretions, including tears, saliva, egg whites, human milk, and gastrointestinal tract (GIT) [6]. Hen egg white contains a high level of Lysozyme enzyme that is thermally stable with a high melting point, reaching 72 °C at pH 5.0 and remaining stable at pH (6–9). It catalyzes the peptidoglycan (PGN) component of the cell envelope of gram-positive microbes by cleaving 1,4-beta-linkages between N-acetylmuramic acid and N-acetyl-D-glucosamine residues, causing lysis of the bacteria [7]. PGN fragments associated with bacterial cell wall lysis are regularly discharged in the gastrointestinal tract and accumulate in the lumen of the intestine [8]. They are recognized as a microbe-associated molecular pattern (MAMP) by immunity cells and activated inflammatory processes [9]. Also, it leads to the production of pro-inflammatory cytokines, antimicrobial peptides, and a protective response to microbes responsible for infection [10,11]. Microbial muramidase hydrolyzes PGN, enhances nutrient absorption, minimizes inflammation, and redirects nutrients for the growth of chickens. The fortification of broilers’ diets with muramidase increases weight gain and improves FCR [7,12]. Also, the supplementation of nursery pigs’ diets with hen egg white muramidase improves the gain rate and the efficiency of feed utilization due to the improvement in small intestine histology [13,14,15] or owing to a strong general immune response [4,5,14]. Muramidase can be a safe feed additive for weaning pigs [13]. Microbial muramidase has recently been investigated for improving intestinal histomorphology, meat quality, immune response, and its hypolipidemic effect in broiler chickens [15]. The current study aimed to evaluate the possible effects of dietary MMUR on the growth parameters, liver histoarchitecture, antioxidant status, biochemical analysis, and the expression of pro-inflammatory cytokines of broiler chickens after a feeding period of 35 days.

## 2. Material and Methods

### 2.1. Feed Additive Used

Microbial muramidase, EC Number 3.2.1.17, lysozyme or N-acetylmuramidase, is produced by fermentation with a genetically modified strain of *Trichoderma reesei* (Accession number DSM 32338), Balancius™, Isando. The enzyme activity in the product is 60,000 LSU(F)/g.

### 2.2. Birds

This trial was undertaken in the Poultry Research Division of the Faculty of Veterinary Medicine at Zagazig University, Egypt. Four hundred one-day-old male Ross-308 chickens were acquired from a native hatchery. On arrival, they were reared on a broad range antibiotic (neomycin) and a dehydrated solution for three successive days and attained an average weight of 97.68 ± 0.59 g. The chicks were nourished in a logically aired house with sawdust litter. The building temperature was adjusted by heaters and was maintained at 34 °C through the initial week and gradually minimized until it reached 25 °C in the last week. The lighting program was 24 h/day for the first week, then changed to 16 h of light and 8 h of darkness in the period of 7–35 days. All chicks were vaccinated against Gumboro and New Castle diseases and checked daily for health problems.

### 2.3. Experimental Design and Diets

The chicks were allotted randomly to 4 groups with ten replicates (100 birds/group). The trial groups comprised of G1: a basal diet without MMUR (control group), G2: a basal diet + 200 mg MMUR kg^−1^, G3: basal diet + 400 mg MMUR kg^−1^, and G4: basal diet + 600 mg MMUR kg^−1^, with an enzyme activity of 0, 12,000, 24,000, and 36,000 LSU(F)/kg diet, respectively. The trial was extended to 35 days with water and feeds offered ad libitum. All feeds were crushed and formulated according to the Ross manual guide published by AVIAGEN [16], as represented in Table 1. Dry matter, crude protein, crude fiber, and fat were analyzed in all feedstuffs and rations used by AOAC [17] The Ca and P content of the diets were measured using an Element Analyzer with an Energy Dispersive X-ray fluorescence system, JSX 3222, JEOL, Japan. The amino acid content of the diets was assessed following the methodology described by Simpson et al. [18], using an amino acid analyzer system (Harvard Bioscience, Inc., Holliston, United States).

### 2.4. Growth Performance

The chicks were weighed separately on the fourth day to determine their initial weights, and the average body weight (BW) from each group was assessed on days 10, 23, and 35. The body weight gain (BWG) was estimated as the difference between the final and the initial weight at each stage interval. The average feed consumption per bird in each replicate was calculated as the difference between the amount of feed offered and the amount of feed left, then divided by the birds’ numbers. The feed conversion ratio (FCR) was determined according to Wagner et al. [19]. FCR = feed intake (g)/body weight gain (g).

### 2.5. Sample Collection

Ten blood samples were taken from the brachial vein of ten random birds from each group (*n* =10) on the 35th day in a dry clean tube without anticoagulant and left to clot at room temperature and then centrifuged at 3000 rpm for 5 min to separate the serum. The collected serum was preserved at −20 °C in Eppendorf tubes prior to analysis.

### 2.6. Clinico-Biochemical Analysis

The serum levels of alanine aminotransferase (ALT) and aspartate aminotransferase (AST) were measured using analytic kits (Spinreact, Santa Coloma, Spain). The glucose levels were assessed with colorimetric investigative kits from Spectrum-Bioscience (Egyptian Company for Biotechnology, Cairo, Egypt), as discussed by [20]. ELISA kits (My Biosource Co. of CAT. NO. MBS269454, MBS265796, MBS266317, and MBS025331) were used for the analysis of triiodothyronine hormone (T3), thyroxin (T4), growth hormone (GH), and leptin.

### 2.7. Immune and Antioxidant Indices

ELISA kits (Cat. No. MBS2024496) were used to measure the serum interleukin 1beta (IL1β) levels. The activities of Malondialdehyde (MDA) (nmol/mL), total antioxidant capacity (TAC) (U/mL), catalase (CAT) (U/mL), and superoxide dismutase (SOD) (U/mL) were measured in the serum following the methodology of Mcdonald and Hultin [21], Rice-Evans and Miller [22], Aebi [23], and Nishikimi et al. [24], respectively.

### 2.8. Histological and Immunohistochemical Examination

Three liver specimens from each group (*n* = 3) were taken and fixated in 10% neutral formalin. Samples were dehydrated with (75–100%) ascending grades of ethanol, located in xylol I and II, and embedded in paraffin. Then, they were sliced into 4 µm slices (cross and longitudinal) by a microtome (Leica RM 2155, England, UK) and stained by hematoxylin and eosin (H and E) [25]. Pictures from each bird in each treatment (25 pictures/group) were taken by an AmScope 5.0 MP microscope digital camera adjusted on (100× and 400× magnification) a high power field. Stained tissues were inspected for pathological changes such as inflammation, degeneration, apoptosis, and necrosis.

The inflammatory response of chickens was explored in the leucocyte populations using transforming growth factor-beta (*TGF-β*) in the liver and spleen. On the 35th day, samples from the liver and spleen (three samples for each group) were taken to observe the expression of *TGF-β*, following the methodology of Saber et al. [26]. Tissue segments were nurtured in an endogenous peroxidase-blocking mixture containing hydrogen peroxide and sodium azide (DAKO peroxidase blocking reagent, Cat. No. S2001). Then, one to two drops of primary monoclonal antibody supersensitive against TGF-β were added (Cat. BAF240, Novus Biologicals, Briarwood Avenue, Centennial, CO, USA); after that, the slides were stained by hematoxylin and examined under a microscope. Finally, they were analyzed for different immune-positive cells using three high power fields (HPFs) using Image J 1.49 software bundled with 64-bit Java 1.8.0_172 (National Institutes of Health, MD, USA) [27].

### 2.9. Statistical Analysis

The results were evaluated by using a one-way analysis of variance (ANOVA) and a SPSS general linear model (SPSS Inc., Chicago, IL, USA) after applying homogeneity and normality among the different groups using Levene and Shapiro–Wilk tests. The linear and quadratic effects of MMUR were exhibited using orthogonal polynomial contrasts. A Tukey test was used to calculate the significance between the mean values and adjusted at a *p* < 0.05 significance level.

The statistical model is given by:

Yik = U + Ti + Eijk

where Yik = observed value of the response variable, U = observed mean for the response variable, Ti = the fixed effect of the treatment group, Eijk = random error.

## 3. Results

### 3.1. Growth Performance

The performance of broilers is shown in Table 2. Throughout the starter period (4th–10th day), the BW, BWG, and FCR were non-significantly different in broilers fed feeds enriched with MMUR at 200 and 400 mg kg^−1^ compared with the control group. Nevertheless, these parameters were significantly enhanced (linear *p* = 0.041, 0.041, and 0.033, respectively) by dietary MMUR at a level of 600 mg kg^−1^. The addition of MMUR at different levels did not affect the feed intake (FI) compared to the control group. During the grower period (11th–23rd day), birds fed on the 600 mg MMUR kg^−1^ diet showed an increase in BW (*p* = 0.018) and BWG (*p* = 0.033) compared to the control group. The FI was indifferent (*p* = 0.790) among all experimental groups. The MMUR levels at 200 and 600 mg kg^−1^ showed an improved FCR (linear *p* = 0.002). Throughout the finisher period (24th–35th day), dietary MMUR at 600 mg kg^−1^ increased the BW (*p* = 0.015) without impacting the BWG, FI, and FCR. The overall performance results showed that the final BW and BWG were linearly increased (*p* = 0.015) in birds fed MMUR-supplemented diets at 600 mg kg^−1^. The overall FCR decreased (*p* = 0.004) with MMUR at 600 mg kg^−1^. The various levels of MMUR addition had no significant effect on the total FI. The ultimate body weight was at its maximum at 600 mg MMUR kg^−1^, while the smallest weight was detected in the control group.

### 3.2. Clinic-Biochemical Indices

The MMUR effect on the blood biochemical indices of the broilers is presented in Table 3. The enrichment of diets with different MMUR levels did not influence the serum values of AST, ALT, and uric acid but linearly increased the creatinine level (*p* = 0.001) compared with the control group. The addition of MMUR at different doses linearly increased the serum levels of T3 (*p* = 0.001), T4 (*p* < 0.001), and growth hormones (*p* < 0.001). MMUR supplementation at all levels did not affect glucose levels compared with the control group. The serum leptin linearly increased (*p* = 0.017) with MMUR addition compared with the control group.

### 3.3. Antioxidant and Inflammatory Responses

The effect of MMUR on serum antioxidant enzyme activities and serum inflammatory indices of the chickens are displayed in Table 4. The TAC, CAT, and SOD activities increased linearly (*p* ≤ 0.001). In contrast, the MDA level decreased either linearly or quadratically in all MMUR-enriched groups compared to the control group. The levels of IL1β and IFN-γ were linearly amplified (*p* ≤ 0.001) in all MMUR treatments. The highest MMUR supplementation level (600 mg/kg) induced the highest values of these parameters.

### 3.4. Histopathological Findings

**Figure 1:**
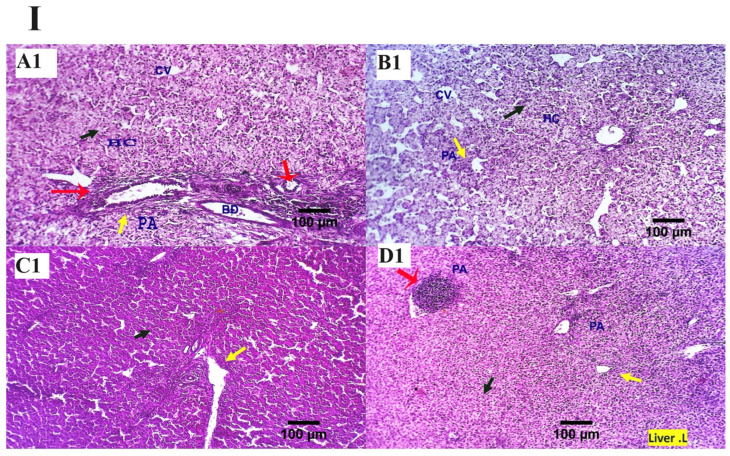

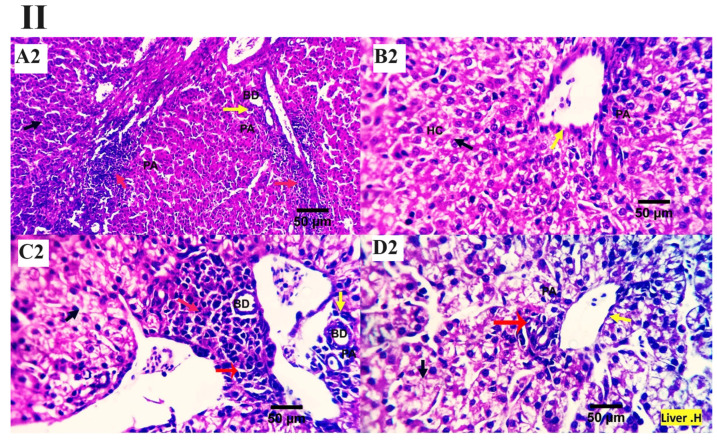
(**I**,**II**) Photomicrographs from the liver ((**I**): H&E × 100 magnification, (**II**): H&E × 400 magnification) of various chicken experimental groups, which received 0 mg/kg MMUR (**A1**,**A2**), 200 mg/kg MMUR (**B1**,**B2**), 400 mg/kg MMUR (**C1**,**C2**), and 600 mg/kg MMUR (**D1**,**D2**). These show typical histological characteristics of various structures, including portal area (PA, yellow arrow) and hepatocytes (HC, black arrow), which are seen as a small masses around the central veins (CV), with a few round cells observed around the portal area indicating a natural immune response. Mild to moderate portal lymphoplasmacytic aggregations (red arrows) and biliary proliferations (BD, yellow arrow) are detected in group (**A**). Mildly aggregated immune-responsive lymphoplasmacytic cells can be observed in groups (**C**,**D**) (red arrows).

The inspected liver sections from the various treated groups (0, 200, 400, 600 mg MMUR kg^−1^) show typical histological characteristics of various structures, including portal area and hepatocytes, which are seen as small masses around the central veins. Several round cells were observed around the hepatic portal area, indicating a natural immune response (Figure 1). Mild to moderate portal lymphoplasmacytic aggregations and biliary proliferation were seen in the control group (Figure 1A). Mildly aggregated immune-responsive lymphoplasmacytic cells were observed for MMUR at levels 400 and 600 mg kg^−1^ (Figure 1C,D). The aggregated inflammatory cells appear to be an immune surveillance and protective device instead of a destructive inflammatory procedure (Figure 1).

### 3.5. TGF-β Immunostaining

**Figure 2:**
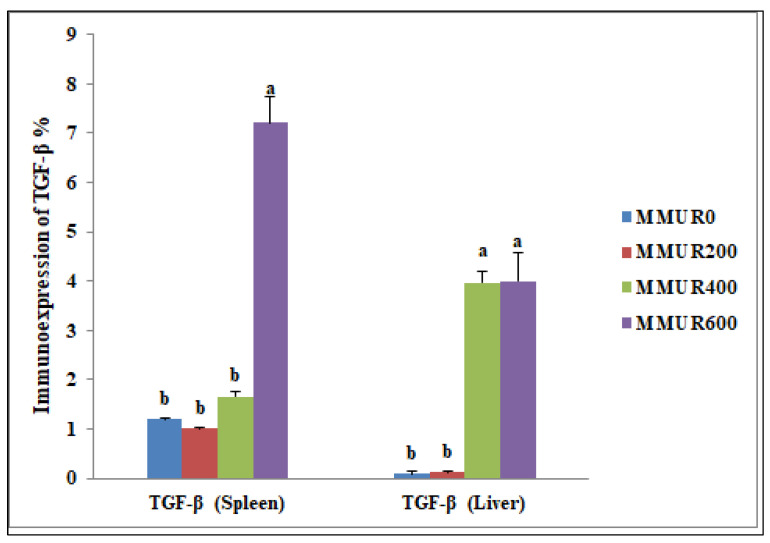
The morphometric analytic data of the immunostained *TGF-β* cells in the spleen and liver of various experimental groups. Bars with different letters (a,b) significantly differ at *p* < 0.05. (MMUR0) group received 0 mg/kg MMUR, (MMUR200) group received 200 mg/kg MMUR, (MMUR400) group received 400 mg/kg MMUR, (MMUR600) group received 600 mg/kg MMUR.

**Figure 3:**
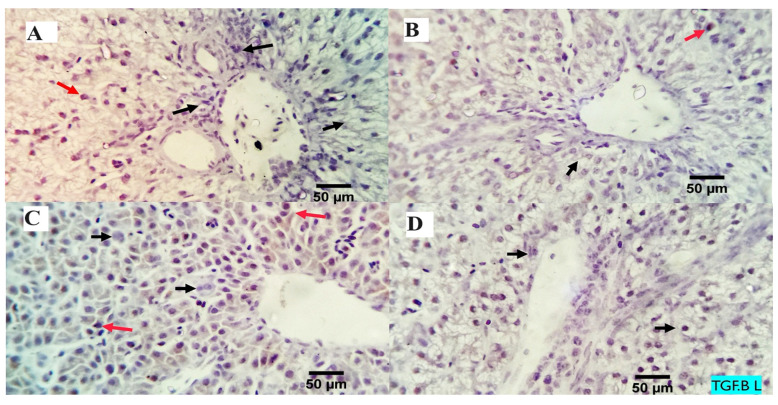
The immunostained positive *TGF-β* cells (red arrows) in the liver of different experimental chicken groups. Black arrows: negative cells. ×400 magnification power. (**A**) group received 0 mg/kg MMUR, (**B**) group received 200 mg/kg MMUR, (**C**) group received 400 mg/kg MMUR, (**D**) group received 600 mg/kg MMUR.

**Figure 4:**
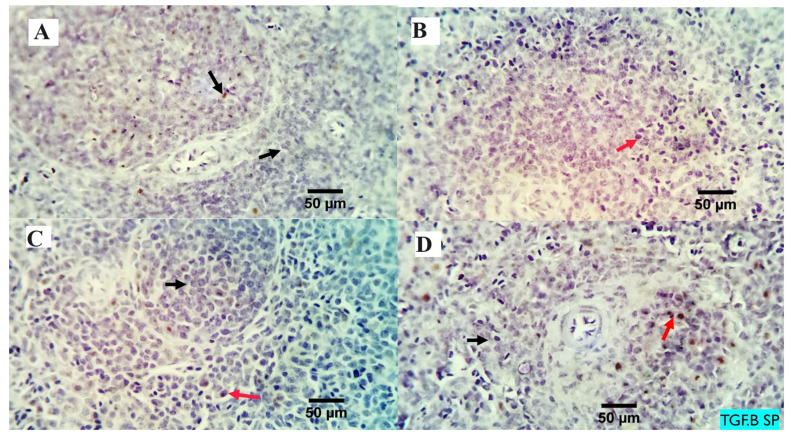
The immunostained positive TGF beta cells (red arrows) in the spleen of different experimental chicken groups. Black arrows: negative cells. × 400 magnification power. (**A**) group received 0 mg/kg MMUR, (**B**) group received 200 mg/kg MMUR, (**C**) group received 400 mg/kg MMUR, (**D**) group received 600 mg/kg MMUR.

The analysis of the examined liver slices exposed an average percentage of positive cells in each of the three high power fields (HPF) to the anti-inflammatory marker (*TGF-β*) as follows: 0.09, 0.13, 3.96, and 4.00 for dietary MMUR levels of 0, 200, 400, 600 mg kg^−1^, respectively (Figure 2 and Figure 3). A morphometric analysis of the inspected spleen segments revealed an average percentage of positive cells in each of the three high power fields (HPFs) to the pro-inflammatory marker (*TGF-β*) in all groups as follows: 1.19, 1.01, 1.66, and 7.21 for dietary MMUR levels of 0, 200, 400, 600 mg kg^−1^, respectively (Figure 2 and Figure 4).

## 4. Discussion

The current study aimed to investigate the impact of dietary supplementation of graded levels of MMUR on the growth performance parameters, liver histoarchitecture, antioxidant status, biochemical analysis, and expression of pro-inflammatory cytokines of broiler chickens. The results showed that adding MMUR to the broilers’ diets encourages the growth performance of broilers’ BW, BWG, and FCR without changing their feed consumption. The highest results were in chickens fed on MMUR at a level of 600 mg/kg, and the lowest was in those fed the control group. This improvement in the performance parameters may be attributed to the excellent condition of the chickens and the improved gastrointestinal tract morphology represented by higher villus length, crypt depth, and absorptive area, as reported in our previous study [15], as well as effective gastrointestinal tract function (good digestion and absorption efficacy) [5], and also the increase in intraepithelial lymphocyte count (IEL), goblet cell numbers, and mucus secretion [5]. Mucus acts as a fence against microbes, physical and chemical attacks, as a lubricant of the GIT tract and its constituents, and as a main component of innate immunity which results in a better feed utilization efficiency [28]. The improved performance caused by MMUR may be due to the increase in the apparent ileal digestibility of dry matter, ether extract, and ash, and decreased intestinal permeability [12,29].

Moreover, MMUR increases the digestibility of fat, protein, and phosphorus [7]. It activates other feed enzymes, such as phytase enzymes [7]. Supplementation with MMUR maintains good intestinal tight junctions, prevents translocation of harmful particles from the lumen of the intestine to the bloodstream, and reduces the exposure of the birds to microbes and their compounds, toxins, and antigens [29,30]. Also, it increases the lactobacillus numbers in broilers’ caecum [12]. Its inclusion with lysozyme maintains healthy gut function, enhances the immune reaction, and also enhances production characteristics in pigs [31,32]. It also minimizes the pathogen counts in broilers’ cecum [32,33], improves growth parameters, and augments antioxidant activity and general immunity of broilers [33].

Previous studies reported a positive effect of microbial muramidase 007 additions to broiler diets on FCR and body weight gain [5,7,12]. Also, the inclusion of MUR in broilers at 35,000 LSU (F)/kg [29] or at 45,000 LSU (F)/kg [34] had an improved effect on body growth and FCR in broilers at 1–42 d. Moreover, muramidase from various origins, for example, the addition of 10% modified rice expressing lysozyme [35] and hen egg white lysozyme [33,36], improved the FCR and BWG of the broilers. On the other hand, previous researchers observed a non-significant effect of hen egg white lysozyme or MMUR inclusion on the performance parameters of broilers [15,37] and pigs [38].

Alanine transaminase (ALT) and aspartate transaminase (AST) are intracellular liver enzymes discharged in high quantities into the blood flow after liver damage. In our experiment, fortifying diets with MMUR at different levels did not affect the serum values of liver enzymes (AST and ALT) and uric acid. However, it increased the creatinine level compared with the control without influencing the serum glucose level. These results confirmed the results of liver histopathology where MMUR supplementation did not cause any harmful effects on the hepatic histoarchitecture, which appeared normal in the non-supplemented groups. Microbial muramidase is an antioxidant that guards cells against the destructive effect of free radicals, lessens toxicity, and maintains liver function [39]. Consistent with our data, Lichtenberg et al. [12] reported no effect of MMUR addition on serum AST value in broilers. Also, Schliffka et al. [38] detected no impact of muramidase addition at 65,000, 325,000, and 650,000 LSU (F)/kg of feed on the serum values of AST, ALT, urea, and glucose in swine. Our results showed that the addition of MMUR to broiler diets resulted in increased values of T3, T4, and growth hormones, which were confirmed by earlier results which stated that the addition of exogenous lysozyme enzyme at 50, 100, and 150 mg/kg of rabbit’s diets increased the serum levels of triiodothyronine and thyroxine [40]. These results also explain the improved growth performance of birds by MMUR supplementation due to the beneficial role of these hormones in controlling growth and other metabolic functions [41].

Lysozyme is an essential ingredient in non-specific humoral immunity. It exerts bactericidal activity by hydrolysis of the β-1,4-glycosidic link between N-acetyl muramic acid and N-acetyl glucosamine of microbial wall peptidoglycans, along with activation of the complement system and phagocytic activity leading to the destruction of the glucosidic bonds of *Escherichia coli* and *Staphylococcus* walls and the prevention of contamination and disease [42]. Our results showed increased serum levels of IL1β and IFN-γ at all MMUR supplementation levels, in addition to the rise in the immunoexpression of *TGF-β* in the liver and spleen tissues by MMUR supplementation in a level-dependent way. Transforming growth factor beta (TGF-β) is a multifunctional cytokine secreted by numerous kinds of cells, including macrophages. It contains three mammalian isoforms (TGF-β 1 to 3, HGNC symbols TGFB1, TGFB2, TGFB3) and several other signaling proteins. The first line of immune response is the release of cytokines, which have a parallel action on the metabolism of nutrients. Pro-inflammatory cytokines forward valuable nutrients far from production and towards the immune reply [43,44]. Cooper et al. [45] documented that TGF-β1 was amplified in unaffected pigs consuming a transgenic goats’ milk lysozyme. The histological examination of the hepatic tissues in the current study showed mildly aggregated immune-responsive lymphoplasmacytic cells at MMUR supplementation levels of 400 and 600 mg kg^−1^. These aggregated inflammatory cells seem to be an immune surveillance and protective device instead of a destructive inflammatory response.

It has been reported that hen egg white lysozyme (HEWL) is highly effective against a wide range of gram-positive microbes, while killing gram-negative microorganisms [46] and minimizing the numbers of *C. perfringens, lactobacilli* and *E. coli* in the ileum of chickens [36]. In addition, the transgenic human lysozyme has activity against numerous pathogens of the digestive tract besides *Clostridium perfringens* [47]. Moreover, it results in the improvement of nonspecific immunity, including the upgrade of the gene appearance of interferon-gamma, interleukin-10 and 18, and the formation of intestinal glutathione peroxidase [4,14,33,37,48]. Our previous study reported linear increases in the serum levels of IL10, complement 3, and lysozyme activity by MMUR supplementation (200–600 mg/kg), in addition to the increase in the immunoexpression of lymphocyte subpopulation biomarkers (CD3 and CD20) in the spleen tissues [15]. Muramidase can split PGN into its ultimate active component, muramyl dipeptide (MDP) [49]. Negroni et al. [50] confirmed that the intracellular nucleotide-binding oligomerization domain-containing protein 2 (NOD2) receptor could sense MDP. Severe stimulation of NOD2 triggers nuclear factor kappa-B cells (NF-κB) and mitogen-activated protein kinase pathways, resulting in cytokine production and a pro-inflammatory response [51].

In the current study, the supplementation of broilers’ diets with MMUR increased the total antioxidant capacity, catalase, and SOD activities while decreasing the level of MDA. The high serum values of TAC, SOD, and GST in broilers fed with MMUR-enriched diets led to the increased ability of the broilers’ antioxidant defense systems to hunt oxidative stress. Exogenous lysozyme is a valuable source of antioxidant compounds that can protect body cells from damage by free radicals and lessen poisonousness [39]. Researchers have previously reported that the enrichment of poultry diets with MMUR has a positive impact on the antioxidant ability of the gastrointestinal tract and increases the gene appearance of intestinal glutathione peroxidase (SOD and GSH-Px) [4,14,33,36,37,48]. Similarly, the addition of the lysozyme enzyme to the diets of rabbits at 50, 100, and 150 mg/kg increased the total antioxidant capacity values, glutathione S-transferase, SOD, and catalase, while decreasing the value of malondialdehyde [52]. Moreover, an increased serum SOD activity and decreased MDA levels of the intestine, accompanied by increased numbers of goblet cells and height of microvilli, were observed following the addition of muramidase to the feeds of juvenile gibel carp [53].

## 5. Conclusions

The enrichment of broiler chicken diets with MMUR at 600 mg/ kg is recommended in practical poultry feeding due to its low cost (8 USD/kg of product at the time of the experiment, while the cost of 600 mg MMUR was 0.48 Cent) and positive influence on the bird’s productive performance, represented by improved body weight, weight gain, and FCR through increased metabolic hormone levels (T3, T4, and growth hormones). Dietary MMUR had no harmful effects on liver tissues and liver function tests. Moreover, it upgraded the antioxidant activity and prevented lipid peroxidation as indicated through increased TAC, catalase, and SOD activities and reduced MDA levels. Dietary MMUR acts as an immune-enhancing substance through increasing IL1β and IFN-γ levels and *TGF-β* immunoexpression in the liver and spleen.

## Figures and Tables

**Table 1:** Proximate and chemical composition of the experimental diets (%).

Ingredients	Starter Period (4–10 d)	Grower Period (11–23 d)	Finisher Period (24–35 d)
Yellow corn	55.86	59.29	62.27
Soybean meal, 48%	33.67	28.085	23.625
Corn gluten, 60%	3.725	5.3	6
Soybean oil	2.2	3	4
Calcium carbonate	1.2	1.2	1.1
Calcium dibasic phosphate	1.5	1.4	1.3
Common salt	0.15	0.15	0.15
Premix *	0.3	0.3	0.3
DL-methionine, 98%	0.4	0.3	0.33
Lysine HCl, 78%	0.47	0.45	0.40
Choline 60%	0.07	0.07	0.07
Threonine	0.1	0.1	0.1
Phytase	0.005	0.005	0.005
Na_2_Co_3_	0.25	0.25	0.25
Anti-mycotoxin	0.1	0.1	0.1
Chemical composition			
ME (Kcal/kg)	3003	3101	3202
Crude protein %	23.04	21.5	20.04
Calcium %	0.941	0.903	0.832
Available phosphorus %	0.482	0.448	0.417
Lysine %	1.47	1.31	1.157
Methionine %	0.721	0.610	0.625
Threonine %	0.825	0.765	0.709

* Premix per kg of diet: Vitamin A, 15,000 IU; Vitamin D3, 2000 IU; Vitamin E, 6500 IU; Vitamin K3, 0.5 mg; thiamine, 1.8 mg; riboflavin, 3.6 mg; pantothenic acid, 10 mg; folic acid, 0.55 mg; pyridoxine, 3.5 mg; niacin, 35 mg; cobalamin, 0.01 mg; biotin, 0.15 mg; Fe, 80 mg; Cu, 8 mg; Mn, 60 mg; Zn, 40 mg; I, 0.35 mg; Se, 0.15 mg.

**Table 2:** Effect of MMUR on the growth performance of broiler chicks during the feeding periods.

Traits	MMUR Level (mg/kg)				*p*-Value		
	0	200	400	600	ANOVA	Linear	Quadratic
Initial BW (g)	98.00 ± 0.001	97.1 ± 0.72	98.1 ± 0.63	97.5 ± 0.001	0.094	0.720	0.611
Starter period (4–10th day)							
BW (g)	332 ± 7.5 ^b^	340 ± 5.9 ^ab^	336 ± 3.1 ^ab^	350 ± 12.3 ^a^	0.103	0.041	0.574
BWG (g)	234 ± 7.5 ^b^	243 ± 6.2 ^ab^	238 ± 3.6 ^ab^	252 ± 12.3 ^a^	0.094	0.041	0.600
FI (g)	268 ± 7.1	265 ± 6.1	264 ± 3.1	265 ± 8.4	0.854	0.520	0.592
FCR	1.15 ± 0.02 ^a^	1.10 ± 0.04 ^ab^	1.11 ± 0.01 ^ab^	1.05 ± 0.07 ^b^	0.094	0.033	0.989
Grower period (11th–23rd day)							
BW (g)	1080 ± 72.5 ^b^	1170 ± 8.7 ^ab^	1182 ± 27.5 ^ab^	1212.62 ± 73 ^a^	0.074	0.018	0.359
BWG (g)	748 ± 65.1 ^b^	830 ± 14.1 ^ab^	846 ± 25.3 ^ab^	862.7 ± 80.6 ^a^	0.119	0.033	0.323
FI (g)	1139 ± 33.2	1147 ± 37.6	1202 ± 47.8	1107.08 ± 94	0.320	0.790	0.165
FCR	1.53 ± 0.09 ^a^	1.38 ± 0.07 ^bc^	1.42 ± 0.03 ^ab^	1.28 ± 0.01 ^c^	0.008	0.002	0.908
4th–23rd day							
BW (g)	1080 ± 72.5 ^b^	1170 ± 8.7 ^ab^	1182 ± 27.5 ^ab^	1212.62 ± 73 ^a^	0.074	0.018	0.359
BWG (g)	982.5 ±55.1 ^b^	1073.4 ± 13.1 ^ab^	1084.44 ± 15.3 ^ab^	1115.12 ± 80.9 ^a^	0.119	0.033	0.323
FI (g)	1405 ± 33.2	1412 ± 37.6	1466 ± 47.8	1371.9 ± 94	0.380	0.752	0.207
FCR	1.43 ± 0.05 ^a^	1.32 ± 0.06 ^bc^	1.35 ± 0.03 ^ab^	1.23 ± 0.01 ^c^	0.008	0.002	0.908
Finisher period (24–35th day)							
BW (g)	1899 ± 78.9 ^b^	2040 ± 54.6 ^ab^	2062 ± 47.2 ^ab^	2131 ± 146.2 ^a^	0.071	0.015	0.511
BWG (g)	819 ± 49.2	869 ± 60.2	879 ± 38.3	918 ± 82.04	0.307	0.080	0.867
FI (g)	1511 ± 32.1	1508 ± 75.1	1560 ± 106.5	1422 ± 137.5	0.418	0.413	0.261
FCR	1.85 ± 0.14	1.74 ± 0.15	1.78 ± 0.19	1.55 ± 0.11	0.173	0.059	0.524
Overall performance (4–35th day)							
Final BW, g	1899 ± 78.9 ^b^	2040 ± 54.6 ^ab^	2062 ± 47.2 ^ab^	2131 ± 146.2 ^a^	0.071	0.015	0.511
Total BWG, g	1801 ± 78.9 ^b^	1943 ± 55.3 ^ab^	1964 ± 46.7 ^ab^	2034 ± 146.2 ^a^	0.071	0.015	0.510
Total FI, g	2919± 68.9	2920 ± 104.9	3027 ± 152.4	2795 ± 239.1	0.396	0.524	0.229
FCR	1.62 ± 0.05 ^a^	1.50 ± 0.07 ^a^	1.54 ± 0.09 ^a^	1.37 ± 0.06 ^b^	0.014	0.004	0.546

^a–c^ Means within the same row carrying different superscripts significantly differ at *p* < 0.05. BW: body weight, BWG: body weight gain, FI: Feed intake, and FCR: feed conversion ratio.

**Table 3:** Effect of MMUR on the blood biochemical parameters of broiler chickens.

Traits	MMUR Level (mg/kg)				*p*-Value		
	0	200	400	600	ANOVA	Linear	Quadratic
ALT (U/L)	5.00 ± 1.00	6.66 ± 1.15	7.33 ± 1.53	7.37 ± 1.53	0.181	0.055	0.307
AST (U/L)	52.0 ± 9.00	54.0 ± 6.25	49.7 ± 2.52	54.0 ± 6.25	0.819	0.922	0.761
Creatinine (mg/dL)	1.99 ± 0.04 ^c^	2.08 ± 0.05 ^b^	2.07 ± 0.04 ^b^	2.15 ± 0.02 ^a^	0.005	0.001	0.883
Uric acid (mg/dL)	2.003 ± 0.12	2.04 ± 0.02	2.05 ± 0.03	2.07 ± 0.04	0.674	0.251	0.898
T3 (ng/mL)	3.34 ± 0.73 ^c^	4.29 ± 0.11 ^b^	4.48 ± 0.15 ^ab^	5.21 ± 0.47 ^a^	0.006	0.001	0.671
T4 (ng/mL)	19.3 ± 0.47 ^c^	22.4 ± 0.80 ^b^	23.31 ± 1.45 ^b^	26.1 ± 1.20 ^a^	<0.001	<0.001	0.829
Growth hormone (ng/mL)	2.63 ± 0.25 ^c^	3.60 ± 0.20 ^b^	4.96 ± 0.21 ^a^	5.20 ± 0.26 ^a^	<0.001	<0.001	0.026
Leptin (ng/mL)	2.01 ± 0.11 ^b^	2.18 ± 0.03 ^ab^	2.22 ± 0.16 ^ab^	2.31 ± 0.13 ^a^	0.081	0.017	0.579
Glucose (mg/dL)	334 ± 5.86	339± 4.58	335 ± 4.73	340 ± 4.04	0.506	0.341	0.908

^a–c^ Mean values in the same row with different superscripts differ significantly (*p <* 0.05). AST: Aspartate aminotransferase; ALT: Alanine aminotransferase.GH: growth hormone, T3: triiodothyronine, and T4: thyroxin hormone.

**Table 4:** Effect of MMUR on antioxidant and pro-inflammatory responses in broiler chickens.

Traits	MMUR Level (mg/kg)				*p*-Value		
	0	200	400	600	ANOVA	Linear	Quadratic
TAC (U/mL)	10.2 ± 0.11 ^c^	11.48 ± 0.99 ^b^	13.1 ± 0.39 ^a^	13.8 ± 0.25 ^a^	<0.001	<0.001	0.388
CAT (U/mL)	2.42 ± 0.52 ^b^	3.55 ± 1.32 ^b^	5.43 ± 0.60 ^a^	6.12 ± 0.93 ^a^	0.004	0.001	0.679
SOD (U/mL)	133± 0.90 ^c^	145 ± 2.91 ^b^	154± 8.69 ^ab^	162 ± 5.29 ^a^	0.001	<0.001	0.540
MDA nmol/mL	5.51 ± 0.45 ^a^	2.89 ± 0.27 ^b^	2.23 ± 0.05 ^b^	2.52 ± 0.53 ^b^	<0.001	<0.001	<0.001
IL1β (ug/mL)	140 ± 4.73 ^c^	153± 13.00 ^bc^	161 ± 3.79 ^ab^	171 ± 3.00 ^a^	0.005	0.001	0.790
IFN-γ (pg/mL)	7.5 ± 2.35 ^c^	11.33 ± 0.96 ^b^	13.6 ± 1.08 ^ab^	14.9 ± 1.13 ^a^	0.001	<0.001	0.174

^a–c^ Mean values in the same row with different superscripts differ significantly (*p <* 0.05). TAC: total antioxidant capacity, CAT: catalase, SOD: superoxide dismutase, MDA: malondialdehyde, IL1β: interleukine1 beta, IFN-γ: interferon-gamma.

## Data Availability

The datasets generated or analyzed during the current study are not publicly available but are available from the corresponding author upon reasonable request.
